# Supplementary material for: Collaborative intelligence in AI: Evaluating the performance of a council of AIs on the USMLE
Source: PLOS Digit Health. 2025 Oct 9;4(10):e0000787. doi: 10.1371/journal.pdig.0000787 (PMC12510544; doi:10.1371/journal.pdig.0000787)
Supplement: S2 File — (ZIP) [file pdig.0000787.s002.zip › S2 Data Raw data files containing unprocessed question inputs and LLM outputs including transcripts of deliberations/STEP3_Question-28-1_Type-MC-NJ.docx]

**Facilitator** **[Round 1]:** A physician is conducting a retrospective review of a trial involving the use of Drug X in patients with a specific disease. It is known that Drug X is associated with an increased probability of cancer in patients who use the drug. A total of 600 individuals with a specific disease were included in the trial. Of the participants, 200 individuals received Drug X and 400 individuals did not receive it. One hundred individuals who received Drug X died of a particular type of cancer and 100 individuals who did not receive the drug died of the same type of cancer. Based on these data, which of the following is the relative risk of death from this type of cancer in individuals who take Drug X as compared with individuals who do not take Drug X? (A) Individuals who take Drug X have an equal risk of dying from this type of cancer (B) Individuals who take Drug X have four times the risk of dying from this type of cancer (C) Individuals who take Drug X have three times the risk of dying from this type of cancer (D) Individuals who take Drug X have two times the risk of dying from this type of cancer (E) The risk for dying cannot be determined from the data

**AI_A:** (1) RESTATING THE QUESTION: The question asks for the calculation of relative risk for death due to a specific type of cancer in individuals that took Drug X compared to those who did not take the drug. Relative risk is a function of epidemiology and it is the ratio of the probability of an event occurring (in this case, dying from a specific cancer) in an exposed group to the probability of the event occurring in a comparison, non-exposed group. (2) PLANNING: The assumptions here are that mortality rates entirely represent the drug's potential risk and that the only difference between the two groups is the use of Drug X. However, this may not be the case as other confounding factors may influence cancer-related mortality. But for this scenario, we can ignore those factors as we don't have any additional information. (3) CLINICAL REASONING: To calculate the relative risk, we need to determine the incidence of death in both groups. The incidence in the exposed group (Drug X) is 100 out of 200 (or 0.50). The incidence in the non-exposed group is 100 out of 400 (or 0.25). (4) CONCLUDING: The relative risk is then calculated by dividing the incidence in the exposed group by the incidence in the non-exposed group, i.e., 0.50/0.25 = 2. (5) RESPONSE: Therefore, the relative risk of death from this type of cancer in individuals who take Drug X compared to individuals who do not take Drug X is two times higher. The correct answer is (D) Individuals who take Drug X have two times the risk of dying from this type of cancer.

**AI_E:** (1) RESTATING THE QUESTION: The query is an assessment of a retrospective trial involving Drug X, used in treating patients with a certain disease. Out of the 600 participating patients, 200 patients were treated with Drug X while 400 patients were not. The trial discovered that 100 patients who took Drug X died of a certain type of cancer, and 100 patients who did not take the drug also died of the same type of cancer. Based on these findings, we are asked to determine the relative risk of death from this specific type of cancer in patients who use Drug X when compared to those who do not use the drug. (2) PLANNING: It is important to understand that the relative risk, in epidemiology, is the ratio of the probability of an outcome in an exposed group to the probability of an outcome in an unexposed group. The "exposed group" here are the patients that received Drug X, and the "unexposed group" are the patients that didn't receive the drug. To compute this, we calculate the proportion of cancer deaths in both groups and compare those proportions. (3) CLINICAL REASONING: By constructing a 2x2 table, we see 100 out of 200 users of Drug X succumbed to cancer, a proportion of 0.5. Similarly, 100 out of 400 non-users died of the same cancer, a proportion of 0.25. Following the formula, the relative risk of death (cancer-specific in this case) due to Drug X, relative to not using Drug X, would be the ratio of those proportions - (0.5/0.25 = 2). (4) CONCLUDING: This step-by-step calculation and comparison of proportions of death in the group of Drug X users versus the non-users shows that patients on Drug X have twice the risk of dying from that specific cancer when treated with the drug. (5) RESPONSE: Therefore, the answer is (D) Individuals who take Drug X have two times the risk of dying from this type of cancer.

**AI_B:** RESTATING THE QUESTION: The query is asking for the relative risk (RR) of dying from a specific cancer for patients who have taken Drug X compared to those who have not. The relative risk is a statistical term used in epidemiological studies to compare the risk of a certain event (in this case, death from a specific cancer) happening in two groups. In this context, Drug X has been associated with an increased probability of cancer. In a trial, 200 participants were given Drug X and 400 were not. Each group had exactly 100 individuals die from a particular type of cancer. PLANNING: In order to calculate the relative risk, we need four values: the number of exposed individuals (people who took Drug X) who experienced the event (death by specific cancer), the total number of exposed, the number of non-exposed individuals (people who did not take Drug X) who experienced the event, and the total number of non-exposed. Since we have all these values, we can go ahead and calculate the relative risk. CLINICAL REASONING: The formula for the relative risk is (a/A) / (c/C), with 'a' representing exposed individuals experiencing the event, 'A' is the total number of exposed individuals, 'c' stands for non-exposed individuals experiencing the event, and 'C' is the total number of non-exposed. Here, 'a' equals 100, 'A' equals 200, 'c' equals 100, and 'C' equals 400. Thus, the relative risk will be (100/200) / (100/400). CONCLUDING: By calculating the values, we get (0.5) / (0.25), which equals 2. RESPONSE: Given these calculations, I conclude that individuals who take Drug X have two times the risk of dying from this specific type of cancer compared to individuals who did not take Drug X. So, the correct option would be (D) Individuals who take Drug X have two times the risk of dying from this type of cancer.

**AI_D:** RESTATING THE QUESTION: In simplified terms, a doctor is revisiting a previous trial in which Drug X was given to patients with a certain disease. It's understood that this drug is linked to a heightened chance of cancer in its users. The trial consisted of 600 participants, 200 were given the drug and 400 weren't. Among the ones who were given Drug X, 100 died due to a form of cancer, similarly, 100 who were not given the drug also died from the same cancer. The question here is, based on this information, what's the relative risk of death due to this cancer for those who take Drug X as compared to those who don't? PLANNING: The key assumptions here involve the understanding of relative risk and mortality rate due to cancer in individuals who take Drug X and those who do not. The principal data involved in this calculation are the numbers of individuals receiving the drug and not receiving the drug, and the numbers of individuals from these groups who died from the same cancer. By questioning these, we might bring into account the relation of cause and effect and the common factors among the diseased. However, from the clinical perspective, we should focus more on the comparative analysis of the drug's effects. CLINICAL REASONING: Relative risk represents the ratio of the probability of an event occurring in the exposed group versus the probability of the event occurring in the non-exposed group. In this case, among those who took Drug X, 100 out of 200 individuals died of cancer, yielding a risk of 0.5. Similarly, in those who didn't take Drug X, 100 out of 400 individuals died of the same cancer, yielding a risk of 0.25. Therefore, the relative risk of death would be the risk in the drug-exposed group divided by the risk in the non-exposed group (0.5/0.25), which equals 2. CONCLUDING: By using step-by-step clinical reasoning, we can surmise from the data given in the question that patients who take Drug X have twice the risk of dying from this particular type of cancer as compared to those who do not take this drug. RESPONSE: So, based on a careful review and comparison of these data, the most accurate option would be (D) Individuals who take Drug X have two times the risk of dying from this type of cancer.

**AI_C:** RESTATING THE QUESTION: So, in other words, the physician is analyzing data from a prior experiment that involved the application of a certain medication, Drug X, on patients suffering from a particular ailment. It's established knowledge that this drug carries the side effect of heightening the risk of cancer. The study encompassed a total of 600 patients with this specific disease; 200 of which were given Drug X, whereas the remaining 400 were not. From those who took the drug, 100 succumbed to a certain kind of cancer, just as 100 of those who did not take the drug met the same fate. What we need to decipher from this data is the relative chance of dying from this type of cancer for those patients who were treated with Drug X as opposed to those who weren't. PLANNING: We need to make certain assumptions here. The rate of cancer-related deaths among those who took Drug X is one possibility and the similar rate among patients who went drug X-free is another. The relative risk would be the ratio of these two proportions. Now, in terms of questioning these assumptions - it's quite probable that other factors outside the trial regarding the use and non-use of Drug X could influence the cancer-related death rates in these patients. Yet in this scenario, we are working strictly based on the provided data and thus this potential assumption would not be relevant to the current discussion. CLINICAL REASONING: The data suggests that of the 200 people who took Drug X, 100 died from the specific type of cancer. Thus the death rate from this specific cancer in the drug-taking group is 100/200 = 0.5 or 50%. In the non-drug-taking group, out of the 400 individuals who did not receive the drug, 100 died of the same cancer. Therefore, the death rate in this group is 100/400 = 0.25 or 25%. CONCLUDING: The relative risk is a ratio of the probabilities or risks in two groups. It is calculated by dividing the probability of an event occurring in the exposed or treatment group (in this case, the group that received Drug X) by the probability of the event occurring in the non-exposed or control group. Here, the relative risk is equal to the risk in the Drug X group divided by the risk in the no drug X group, i.e., 0.5/0.25 = 2. RESPONSE: Hence, based on the data, one could conclude that individuals who take Drug X have two times the risk of dying from this type of cancer compared with individuals who do not take Drug X. The correct answer is (D).

**Facilitator** The question asks for the relative risk of death from a specific type of cancer in individuals who took Drug X compared to those who did not. Relative risk is a statistical measure used in epidemiological studies to compare the risk of a certain event happening in two groups. In this case, the event is death from a specific cancer. The trial involved 600 participants, 200 of whom were given Drug X and 400 were not. Each group had exactly 100 individuals die from the specific type of cancer. To calculate the relative risk, we need to determine the incidence of death in both groups. The incidence in the exposed group (those who took Drug X) is 100 out of 200 (or 0.50). The incidence in the non-exposed group (those who did not take Drug X) is 100 out of 400 (or 0.25). The relative risk is then calculated by dividing the incidence in the exposed group by the incidence in the non-exposed group, i.e., 0.50/0.25 = 2. Therefore, the relative risk of death from this type of cancer in individuals who take Drug X compared to individuals who do not take Drug X is two times higher. The correct answer is (D) Individuals who take Drug X have two times the risk of dying from this type of cancer. It's important to note that this calculation assumes that mortality rates entirely represent the drug's potential risk and that the only difference between the two groups is the use of Drug X. Other confounding factors may influence cancer-related mortality, but for this scenario, we can ignore those factors as we don't have any additional information.
